# First person – Meagan S. Siehr

**DOI:** 10.1242/dmm.044552

**Published:** 2020-03-30

## Abstract

First Person is a series of interviews with the first authors of a selection of papers published in Disease Models & Mechanisms, helping early-career researchers promote themselves alongside their papers. Meagan S. Siehr is first author on ‘ *Arx* expansion mutation perturbs cortical development by augmenting apoptosis without activating innate immunity in a mouse model of X-linked infantile spasms syndrome’, published in DMM. Meagan conducted the research described in this article while a predoctoral fellow in Jeffrey L. Noebels's lab at the Department of Molecular and Human Genetics, Baylor College of Medicine, Houston, TX, USA. She is now a postdoctoral associate in the lab of Jeffrey L. Noebels at the Department of Neurology, Baylor College of Medicine, investigating translational approaches to model catastrophic developmental epilepsies and utilizing these models to understand therapeutic mechanisms.


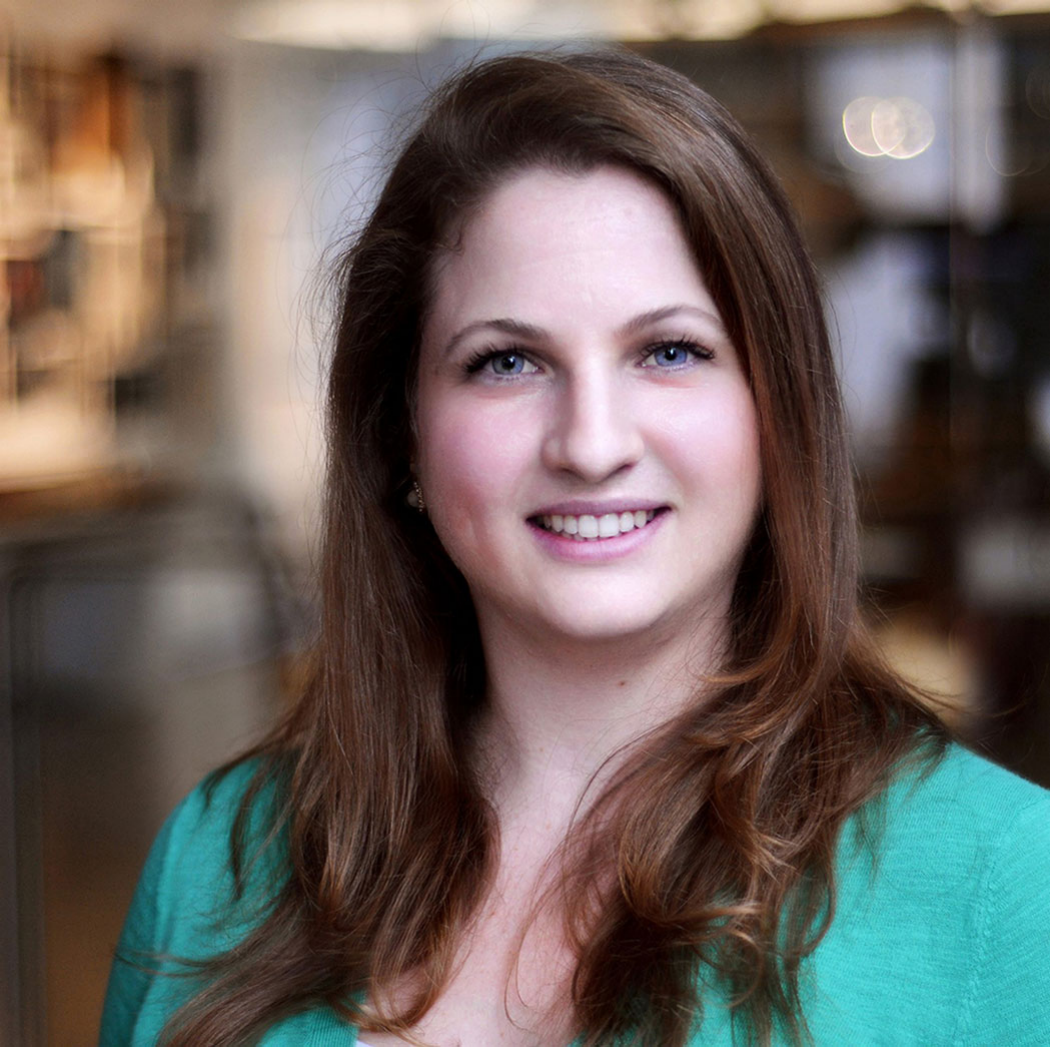


**Meagan S. Siehr**

**How would you explain the main findings of your paper to non-scientific family and friends?**

In this work, we utilized a mouse that harbors a particular mutation in the gene *Arx*, which in human patients causes infantile spasms, a severe, developmental epilepsy syndrome. The *Arx* gene is important for the development of a class of neurons (GABAergic interneurons) that are crucial for modulating brain circuits. We examined early brain development to understand how this *Arx* mutation perturbs neonatal brain development and, moreover, understand how clinical and preclinical hormone therapies [adrenocorticotropic hormone (ACTH) and estradiol] act on the brain during this early period. We found that, during the neonatal period, there is an elevation of cell death that surprisingly does not affect cells that harbor the *Arx* mutation. Interestingly, neither treatment, ACTH nor estradiol, ameliorated increased cell death but they did elevate the number of cells that express *Arx*. Although we were unable to identify the cell type that is undergoing cell death, we speculated that the reason cells may be dying is brain inflammation, as clinical treatment ACTH targets inflammation and it is well-documented that brain inflammation leads to increased cell death. However, we found no evidence of inflammation in the developing brains of mutant mice, which may suggest that the mechanism by which ACTH acts in patients with this type of *Arx* mutation may be due to other mechanisms rather than targeting inflammation.

**What are the potential implications of these results for your field of research?**

We did not observe a therapeutic effect of ACTH in our model; however, many cases of infantile spasms are resistant to ACTH treatment. This *Arx* model may be a model of ACTH-pharmacoresistant epilepsy and a useful tool when testing novel therapies. The exact therapeutic mechanism of ACTH in infantile spasms is unknown. However, we did find that this clinical ACTH treatment, as well as preclinical treatment estradiol, elevated Arx-expressing GABAergic interneurons. It's possible that modulating GABAergic interneurons in the brain may be a potential therapeutic mechanism of this treatment and its role in disorders characterized by loss or defect of these neurons, and its complex actions in different etiologies of infantile spasms should be explored. Neither therapy targeted cell death, highlighting the need for treatments that target all aspects of pathology, cellular and otherwise, in infantile spasms. Currently, there is no evidence that human patients with infantile spasms exhibit abnormal brain cell death, but, due to the catastrophic and debilitating nature of this disorder, this should be explored as it may contribute to epilepsy and comorbidities in this disorder.

**What are the main advantages and drawbacks of the model system you have used as it relates to the disease you are investigating?**

As with many other genetic mouse models of human disease, the *Arx* polyalanine expansion model of infantile spasms has its advantages and disadvantages. The polyalanine expansion mutation in this model recapitulates a human mutation in this gene. In terms of modeling disease, this model has many of the clinical features seen in human patients with this disorder including neonatal spasms (that wane with age as in human patients), seizures and interictal discharges in electroencephalogram (EEG). In terms of testing novel and existing therapies, these phenotypic features make this model ideal. However, modeling epilepsy in an animal model has its challenges. Seizures and spasms in genetic animal models of epilepsy are inherently stochastic and typically have variable expressivity or incomplete penetrance. Conducting drug studies with these models is difficult and requires large samples and long EEG recording sessions. Regardless, mouse models such as ours and other epilepsy models are invaluable to drug discovery.

**Figure:**
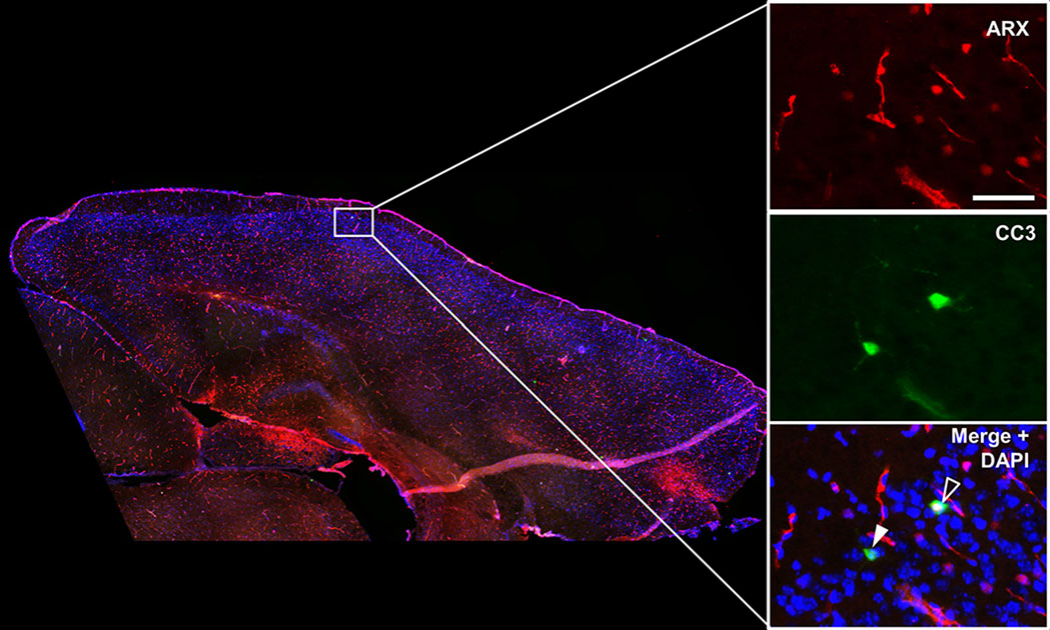
**Immunohistochemistry on a brain slice of a neonatal *Arx* mutant, a genetic model of infantile spasms.** Arx protein is shown in red and the apoptotic marker cleaved caspase-3 (CC3) is shown in green. The filled arrowhead indicates an apoptotic cell in the cortex and the open arrowhead indicates an apoptotic cell that expresses Arx protein.

**What has surprised you the most while conducting your research?**

What has surprised me most is the crucial need for preclinical data and discovery of novel treatments in the catastrophic developmental epilepsies. Often patients are resistant to current therapies, and there is an immense need for developing novel therapies for these disorders. I and my colleagues have been approached by both clinicians and families asking about putative therapies for their patients and children because they have not been able to achieve seizure freedom or any therapeutic effect with current therapies. In addition, many of the genetic perturbations found in these patients are rare mutations in genes that little is known about. Thus, there is an immense need for patient-derived models that recapitulate these mutations in mouse or cell-based models.

**Describe what you think is the most significant challenge impacting your research at this time and how will this be addressed over the next 10 years?**

A significant challenge currently impacting our research is being able to effectively untangle brain complexity in the developing cortex. Pinning down which cell types are undergoing exaggerated cell death in our *Arx* model is a huge challenge. Techniques utilizing single-cell sequencing such as Drop sequencing could be useful but they do have limitations when it comes to adequately capturing apoptotic cells, as the number of apoptotic cells in the cortex make up a very small percentage compared to the total cell population. New, high-throughput and highly sensitive assays are required to determine the identity of these dying cells.

“[…] improving connections and communication between the clinic, patients, research organizations and researchers would greatly improve the professional lives of early-career scientists focused on understanding the underlying etiologies of human disease.”

**What changes do you think could improve the professional lives of early-career scientists?**

I firmly believe that improving connections and communication between the clinic, patients, research organizations and researchers would greatly improve the professional lives of early-career scientists focused on understanding the underlying etiologies of human disease. Within the scope of genetic disorders, determining the effects of novel mutations requires collaboration between researchers, clinicians and the patients that suffer from these debilitating mutations. Connecting with clinicians, patients and their families provides novel avenues for early-career scientists as it generates new hypotheses regarding mechanisms underlying genetic disorders. Most importantly, these collaborative efforts can push disease-modifying treatments to the forefront and ultimately impact patient quality of life.

**What's next for you?**

My passion for translational research has taken me on a less canonical journey into the field of genetic counseling. I began my scientific training working with genetic models in worms and mice, and I am currently working on a project understanding the genetic underpinnings of sudden unexpected death in epilepsy (SUDEP). I feel that the best use of my training in genetics is to apply it to clinical practice and guide patients along their path to diagnosis. In addition, I'd like to continue clinical research in rare genetic disorders such as infantile spasms and SUDEP.
